# Novel layers of RNA polymerase III control affecting tRNA gene transcription in eukaryotes

**DOI:** 10.1098/rsob.170001

**Published:** 2017-02-22

**Authors:** Ewa Leśniewska, Magdalena Boguta

**Affiliations:** Department of Genetics, Institute of Biochemistry and Biophysics, Polish Academy of Sciences, Pawińskiego 5a, 02-106 Warsaw, Poland

**Keywords:** tRNA, Pol III, TFIIIC, polymerase assembly, transcription elongation, transcription termination read-through

## Abstract

RNA polymerase III (Pol III) transcribes a limited set of short genes in eukaryotes producing abundant small RNAs, mostly tRNA. The originally defined yeast Pol III transcriptome appears to be expanding owing to the application of new methods. Also, several factors required for assembly and nuclear import of Pol III complex have been identified recently. Models of Pol III based on cryo-electron microscopy reconstructions of distinct Pol III conformations reveal unique features distinguishing Pol III from other polymerases. Novel concepts concerning Pol III functioning involve recruitment of general Pol III-specific transcription factors and distinctive mechanisms of transcription initiation, elongation and termination. Despite the short length of Pol III transcription units, mapping of transcriptionally active Pol III with nucleotide resolution has revealed strikingly uneven polymerase distribution along all genes. This may be related, at least in part, to the transcription factors bound at the internal promoter regions. Pol III uses also a specific negative regulator, Maf1, which binds to polymerase under stress conditions; however, a subset of Pol III genes is not controlled by Maf1. Among other RNA polymerases, Pol III machinery represents unique features related to a short transcript length and high transcription efficiency.

## 1. Introduction

Transcription of nuclear DNA in eukaryotes is carried out by at least three different RNA polymerases (Pols), designated Pol I, II and III. Each RNA Pol catalyses the transcription of a specific set of genes. The set of transcripts synthesized by Pol II is extremely complex, because it includes all the different protein-coding mRNAs (from several thousand to tens of thousands in different eukaryotes) and many non-protein-coding RNAs, such as snRNAs, snoRNAs and micro (mi)RNAs. By contrast, Pol I and Pol III are specialized in high-level synthesis of protein-non-coding RNA species, rRNA and tRNA, which are fundamental components of the translation machinery. tRNA and rRNA genes are highly transcribed, leading to the production in yeast of 3 million tRNAs per generation and 300 000 ribosomes, compared with about 60 000 molecules of mRNA.

Within the past decade, substantial progress has been made to understand the unique features of Pol III transcription machinery. This review gives a comprehensive overview of the mechanisms, which have potential impact on the levels of Pol III transcripts. We concentrate mostly on regulation of tRNA synthesis in budding yeast, the simplest and well-recognized model of the eukaryotic cell. Starting from the biogenesis of Pol III complex, we describe promoter recognition by Pol III general factors and the cascade of DNA–protein interactions leading to recruitment of Pol III to their genes. In the context of the recently solved structure of Pol III complex and genome-wide analysis of actively transcribing enzyme, we discuss the mechanisms of transcription initiation, elongation and termination. Finally, we summarize the data on Pol III control by Maf1, a general negative regulator, and by phosphorylation of Pol III subunits.

Several interesting aspects of Pol III regulation are, however, beyond the scope of this review. One topic which is not covered is the chromatin connections of Pol III-transcribed genes, but there is an excellent review available on the subject [1]. Another aspect of Pol III control not discussed here is non-uniform regulation of tRNA genes that can shift the translation profiles of key codon-biased mRNAs. For this topic, the readers may be referred to other recent reviews [2,3].

## 2. Biogenesis of RNA polymerase III

Pol III, composed of 17 subunits of total mass approximately 700 kDa, is the largest of the three Pols in yeast. An atomic model of the yeast Pol III elongation complex has been built by reconstruction of the cryo-electron microscopy structure at 3.9 Å resolution [4]. The structural core of Pol III consists of 10 subunits: C160 and C128, forming the active-centre cleft; AC40 and AC19, which are common between Pol III and Pol I; C11, involved in transcription termination; and five small subunits, ABC27, ABC23, ABC14.5, ABC10β and ABC10α, shared between Pol I, Pol II and Pol III. On the periphery of the core enzyme are additional subunits, which form Pol III-specific subcomplexes, C82-C34-C31 and C53-C37, which function in transcription initiation and termination. Additionally, C17 and C25 form a Pol III stalk involved in transcription initiation [5]. Pol III enzymes are highly conserved between organisms. The structure and biogenesis of Pol III are studied mostly in yeast; the exception is structural analysis of Rpc32β-Rpc62 subcomplex of human Pol III [6].

The larger subunits of Pol III core are conserved in sequence, structure and function. C160 and C128 are related to the β’ and β components of α_2_ββ′ω bacterial RNA polymerase, and AC40 with AC19 have local similarities to bacterial α subunits [7].

A hypothetical model of Pol III assembly is based on the relatively well-recognized analogous process for prokaryotic RNA polymerase. It starts with the formation of the αα dimer [8], which interacts with β subunit [9], and then β′ subunit is recruited [10]. The existence of intermediate complexes in the process of Pol III assembly is suggested by mass spectrometry analysis of Pol III disassembly. This analysis has revealed stable subcomplexes C128-AC40-AC19-ABC10β-ABC10α (analogue of ααβ bacterial core subcomplex) and C160-ABC14.5-ABC27 (β′—like module), suggesting their formation in the initial step of complex assembly [11]. The relatively easy *in vitro* dissociation of C25-C17, C37-C53 and C82-C34-C31 modules from Pol III suggests that the peripheral subunits are added as Pol III-specific subcomplexes later in the Pol III assembly [11,12].

Numerous studies on Pol II complex biogenesis (reviewed in [13]) have led to a model in which Pol II is assembled in the cytoplasm with the help of assembly factors and transported to the nucleus as a complex together with a specific adaptor which, following dissociation from Pol II in the nucleus, is exported back to the cytoplasm. Probably, Pol I and Pol III core enzymes use a similar assembly pathway [13].

As Pol III functions in the nucleus, as do other Pols, all 17 of its subunits must be imported to the nucleus, either individually or as part of larger multisubunit assemblies. Nuclear import of a protein requires a nuclear localization signal (NLS) within its sequence but among the Pol III subunits only C128 has a weak NLS. Remarkably, deletion of the NLS-containing region in C128 brings about cytoplasmic localization not only in C128 itself, but also some other Pol III subunits (C160, C53 and C11), without affecting the nuclear localization of C25, C82 and AC40 [14].

**Figure 1.**
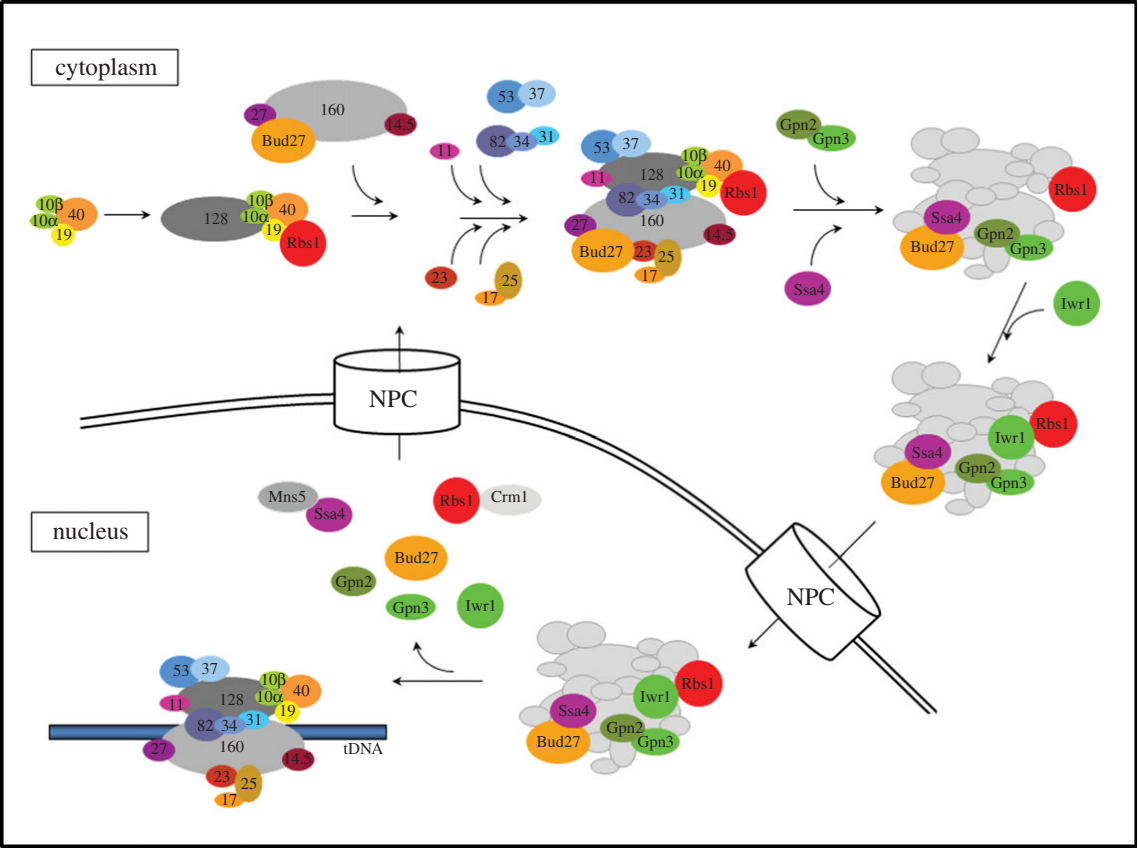
Pol III biogenesis. Based on the relatively well-studied analogous process for prokaryotic RNA polymerase, it is postulated that the assembly of yeast Pol III starts with the formation of the AC19/AC40 subcomplex, probably together with the small ABC10β/ABC10α subunits, which then binds the second-largest catalytic subunit C128. The stable subcomplex C128/AC40/AC19/ABC10β/ABC10α binds the Rbs1 factor via AC40 and AC19. In a parallel step, the second major assembly intermediate is formed by the largest subunit, C160, and the ABC27 and ABC23 subunits incorporated with the help of Bud27. Pol III core is formed by joining of the two subcomplexes. Then the peripheral subunits are added as Pol III-specific subcomplexes (once the Pol III holoenzyme is assembled, Pol III subunits are presented in grey, for clarity). Gpn2, Gpn3 and Ssa4 presumably participate in later steps of Pol III biogenesis, and Iwr1 acts downstream of the GTPases and Ssa4. According to the presented model, Pol III complex is assembled in the cytoplasm prior to the nuclear import. It is also conceivable that only the core complex is formed in the cytoplasm and the peripheral subunits join it in the nucleus, as discussed in the text. Pol III is imported into the nucleus via the nuclear pore complex (NPC), probably together with the adaptors and assembly factors. The transport/assembly factors dissociate from Pol III and are exported back to the cytoplasm; Rbs1 and Ssa4 are exported, respectively, in Crm1- and Msn5-dependent manner.

This again suggests that the Pol III core could be assembled in the cytoplasm, whereas additional complexes, in particular C17–C25 and C82–C34–C31, would only bind the core in the nucleus [14]. It is therefore likely that besides factors common to all three Pols, the assembly of Pol III requires specific auxiliary proteins (figure 1).

One candidate Pol III assembly factor is Bud27, an unconventional prefoldin protein, which contains both NLS and NES (nuclear export signal) sequences, and shuttles between nucleus and cytoplasm [15]. As shown by co-immunoprecipitation and mass spectrometry, Bud27 interacts directly with some subunits of Pol III (C160, C128, C25, AC40, ABC27 and ABC10β), plays a role in Pol III assembly and may serve as a shuttling adaptor for nuclear transport of Pol III [15,16]. It is also required for proper incorporation of the ABC27 and ABC23 subunits to all three RNA Pols [15] (figure 1).

Another candidate is the Rbs1 protein identified by genetic suppression of a missense mutant defective in Pol III assembly [17]. Reduced interactions between subunits of the complex in the assembly Pol III mutant were corrected by overproduction of Rbs1. Rbs1 was found experimentally to interact with AC40, AC19 and ABC27 subunits [17]. Additionally, Rbs1 interacts with the exportin Crm1, and shuttles between the cytoplasm and the nucleus (figure 1). All these data suggest that Rbs1 binds to Pol III holocomplex or a subcomplex, facilitates its translocation to the nucleus and is exported back to the cytoplasm by Crm1 [17].

Gpn2 and Gpn3, members of a poorly characterized but evolutionarily conserved family of small GTPases, could also be involved in Pol III biogenesis. *GPN2* and *GPN3* mutants are defective in nuclear localization of Pol III subunits C53 and C160 [18] (figure 1).

Ssa4, a heat shock protein of Hsp70 family, which also shuttles between nucleus and cytoplasm, is yet another player in Pol III biogenesis. It interacts with C160 in a Bud27-dependent manner, and in *ssa4Δ* cells C160 is partially mislocalized to the cytoplasm [16]. The Ssa4 export from the nucleus requires the Msn5 exportin [19] and *MSN5* deletion resulted in partial mislocalization of C160 to the cytoplasm because of nuclear accumulation of Ssa4 [16] (figure 1).

The last putative Pol III assembly/import factor, Iwr1, contains an NLS in the N-terminal region and was initially implicated in the nuclear import of Pol II [20]. However, further studies have revealed that Iwr1 interacts weakly with C160 [21] and *iwr1Δ* strains are defective in nuclear localization of other Pol III subunits (C53, C37, C160 and AC40) [18]. According to the proposed model, Iwr1 acts downstream of the GTPases involved in the assembly of both Pol II and Pol III [18] (figure 1). Interestingly, Iwr1 also plays an important role in preinitiation complex formation by all three nuclear RNA Pols in yeast [21]. Another interesting link between polymerase assembly and transcription regulation is sumoylation of C82 subunit important for interaction between subunits but also required for efficient transcription of Pol III genes in optimal growth conditions [22].

## 3. Pol III transcriptome

**Figure 2.**
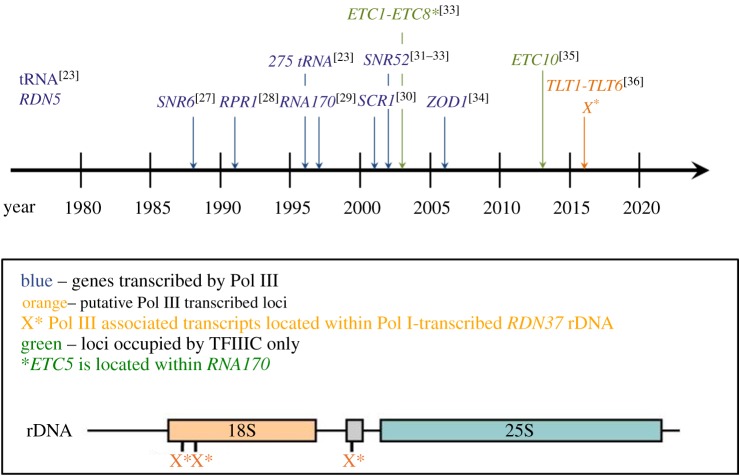
Historical view of Pol III-transcribed genes. The timeline presents approximate dates of identification of *S. cerevisiae* Pol III-transcribed loci as well as loci occupied only by TFIIIC. Numbers in superscript refer to the respective publications.

The history of identification of the yeast genes transcribed by Pol III is summarized in figure 2. It has long been known that Pol III transcribes tRNA and 5S rRNA. Sequencing of the *Saccharomyces cerevisiae* genome has revealed 275 nuclear genes encoding tRNA, which are dispersed on all chromosomes. By several criteria, all yeast tRNA genes can be considered active [23]. The same set of genes, including tRNA gene tX(XXX)D of unknown specificity, but very similar to serine tRNA, was predicted using search algorithms, such as Pol3scan or tRNAscan-SE, based on consensus sequence motifs inside tRNA genes [24–26]. The length of tRNA genes varies between 72 and 133 nt, and only a minority of them (61 genes) have an intron. The primary transcripts of all tRNA genes must undergo maturation at both ends and, when needed, intron excision to generate mature tRNA. Yeast tRNA genes are grouped into 42 families of distinct codon specificity [25,26].

Other short protein-non-coding RNAs, detected as Pol III transcripts a long time ago, include *SNR6*-encoded U6 snRNA, which mediates catalysis of pre-mRNA splicing and the RNA component of RNase P involved in maturation of tRNA primary transcripts encoded by *RPR1* [27,28]. Later RNA170 of unknown function and the RNA subunit of signal recognition particle (SRP) encoded by *SCR1* gene were identified as Pol III transcripts [29,30]. First attempts at genome-wide identification of the yeast Pol III transcriptome employed chromatin immunoprecipitation (ChIP) [31–33]. In three independent analyses performed in different laboratories, all tRNA genes were found to be occupied by Pol III and general transcription factors TFIIIB and TFIIIC, but the absolute levels of occupancy varied among them. It was therefore inferred that all yeast tRNA genes are actively transcribed, but with different efficiency. Essential components of the Pol III machinery were also identified on the *SNR52* gene encoding snR52 snoRNA, which serves as a methylation guide for rRNA and was further confirmed as a Pol III transcript [31–33]. Moreover, two loci, *YGR033C* and *YML089C*, were occupied by all three components of the Pol III machinery, and four others, *YGR258C*, *YOR228C*, *YBR154C* and *YOL141W*, by TFIIIC only [32]. A region near *YML089C* was also occupied by all three components of Pol III machinery and was named zone of disparity (*ZOD1*) [33]. It was shown later that *ZOD1* is an ancient gene for tRNA-Ile and is weakly transcribed by Pol III [34]. Similarly, *YGR033C* derives from a tDNA-Arg ancestor [34]. Another analysis identified eight loci occupied only by TFIIIC, called *ETC1-8* for extra TFIIIC [33]. Probably, the occupancy by TFIIIC included the four loci listed above and the slightly different assignments were due to low resolution of ChIP [32,33]. Then, *ETC10* (region between *MPD1* and *YOR289W*) was identified as an extra TFIIIC site [35]. *ETC5* is part of the *RNA170* gene. In standard growth condition, RNA170 is weakly detectable, but its expression increases dramatically after nucleosome depletion or after changing carbon source to a non-fermentable one and elevating temperature to 37°C [34,36]. The increased expression of *RNA170* and *ZOD1* upon nucleosome depletion is not paralleled by a proportional increase in occupancy by the transcription machinery [34]. Those authors suggest therefore that the derepression of *ZOD1* and *RNA170* transcription upon nucleosome depletion involves activation of poised Pol III.

Presently, transcriptomes are defined and investigated using next generation sequencing (NGS)-based approaches. The human Pol III transcriptome verified by the ChIP-seq method contains 522 predicted tRNA genes and 109 pseudogenes [37]. In contrast, with the yeast genome, where all tRNA genes reside in a nucleosome-free region, only about half of the tRNA genes are Pol III-occupied or nucleosome-free in higher eukaryotes. Besides tRNAs, 5S rRNA, U6 snRNA and 7 SL RNA, human Pol III also transcribes short interspersed nuclear elements SINEs, 7 SK RNA, RNase MRP RNA, vault RNAs and Y RNAs [37,38].

Two novel techniques employed for studies on eukaryotic transcriptomes are native elongating transcript sequencing (NET-seq) [39–41] and the UV cross-linking and analysis of cDNA (CRAC) [36]. Both CRAC and NET-seq provide single nucleotide resolution, and CRAC was used earlier to identify yeast transcripts bound by nuclear RNA surveillance factors [42]. For the yeast Pol III transcriptome, CRAC confirmed the association of Pol III with the previously known Pol III transcripts and revealed six potential new ones called *TLT* (tRNA-like transcripts). Expression of *TLT1* and *TLT6* loci was confirmed by Northern hybridization. The function of these transcripts is so far unknown. Interestingly, distinct Pol III-associated transcripts were located within the Pol I-transcribed *RDN37* rDNA [36]. The coexistence of Pol I and Pol III in the region of 18S rDNA deserves future studies. Moreover, numerous non-coding RNA that are generally transcribed by Pol II showed greater than twofold increase transcription by Pol III under stress conditions [36]. These results suggest that upon stress some Pol II transcripts are increasingly transcribed by Pol III. The development of new, more sensitive techniques and the already noted increased expression of some genes in other than standard conditions (e.g. *RNA170* and *ZOD1*) provide open questions concerning the Pol III transcriptome in the simplest eukaryotic cell.

## 4. Recognition of Pol III promoters by TFIIIC

Unlike Pol II, the Pol III machinery recognizes conserved promoter elements located within the transcribed region. In most Pol III genes, these are the so-called box A and box B sequences, which at the RNA level contribute to the universally conserved D- and T-loops in the tRNA structure. The internally located A- and B-boxes are the main *cis*-acting control elements for transcription of tRNA genes (with the exception of the selenocysteine tRNA gene). In 61 tRNA genes, the A- and B-boxes are separated by an intron; therefore, the distance between these two promoter elements varies from 31 to 93 nt. Assuming that the 5′-end of mature tRNA corresponds to position ‘0’, the A-box starts at position +8 downstream and the transcription start site is usually located between 10 and 12 nt upstream. In *SCR1,* the longest Pol III gene in *S. cerevisiae*, the A- and B-boxes are also located in the region encoding the mature transcript, whereas in the *RPR1* and *SNR52* genes these promoter elements sit in 5′ leader sequences. By contrast, the *SNR6* gene promoter comprises a TATA box upstream of the transcription start site, the A-box in the coding region and the B-box in the 3′ trailer [38]. Finally, the *RDN5* gene, present in multiple copies, contains the A-box, an intermediate element and the C-box, all located in the transcribed region.

The conserved promoter elements in DNA are recognized by the general transcription factors specific to Pol III. The A- and B-box together form a bipartite binding site for the six-subunit basal transcription factor TFIIIC. Recruitment of TFIIIC to the *RDN5* gene, lacking the B-box, is dependent on TFIIIA factor, which binds to the C-box and acts as an adaptor [43]. The association of TFIIIC with DNA initiates a cascade of DNA–protein interactions: TFIIIC-directed recruitment and assembly of the three subunits of the TFIIIB factor and subsequent recruitment of the Pol III enzyme to the transcription start site. Pol III genes are generally short and transcription terminates on a stretch of T-residues variably located less than 20 nt from the 3′-end of the mature RNA.

TFIIIC is composed of two subcomplexes, τA and τB, connected by a linker. Owing to its naturally elastic structure, TFIIIC can cope with the variable distance between A- and B-boxes in Pol III genes, allowing their binding by τA and τB, respectively [35]. The main determinant of both the selectivity and stability of TFIIIC–DNA complexes is the τB binding to the B-box whereas the A-box involvement in transcription initiation is more subtle [44]. The τB module comprises τ138 (Tfc3), τ91 (Tfc6) and τ60 (Tfc8), while τA is composed of τ131 (Tfc4), τ95(Tfc1) and τ55 (Tfc7). Although only Tfc1 and Tfc3 bind DNA directly, all six subunits of TFIIIC are essential *in vivo*. Tfc4 contains an N-terminal TPR array domain, which binds an unstructured, central region of Tfc3 providing the τA-τB linker within TFIIIC complex [45]. The transcription termination region in Pol III genes is flexibly accommodated within the TFIIIC–DNA complex regardless of variable distance from B-box, which explains why the whole gene sequence is protected by TFIIIC; moreover, this interaction delimits the 3′-boundary of the transcription unit [35].

## 5. Role of TFIIIC in recruitment of general transcription factor TFIIIB

Transcription initiation is regulated by TFIIIC-dependent recruitment of TFIIIB factor composed of three subunits. Early genetic and biochemical studies (reviewed in [44]) suggested that the Tfc4 subunit of TFIIIC, positioned upstream of the transcription start site, recruits two subunits of TFIIIB, Brf1 and Bdp1, whereas the Tfc8 subunit of TFIIIC interacts with the third subunit of TFIIIB, TBP. More recently, high-resolution structure determination revealed distinct regions of Brf1 and Bdp1 binding on Tfc4 [45]. Importantly, the site of Bdp1 interaction overlaps that of Tfc3, resulting in binding competition. As a consequence, Tfc4 of the τA module cannot simultaneously recruit Bdp1 and form the linker with τB module by its interaction with Tfc3 subunit. According to the proposed model [45], the assembly of TFIIIB is initiated by the recruitment of Brf1 to Tfc4, probably by the completed assembly of TFIIIC on a tRNA gene. The second TFIIIB component, namely TBP, is then recruited via binding sites on Brf1 and via the Tfc8 subunit. The final step of TFIIIB recruitment is Bdp1 binding to Tfc4, which, however, requires dissociation of Tfc3 and the displacement of the τB module, causing a conformational change of the TFIIIC complex. This model, in which Bdp1 induces the displacement of the τB module as a regulatory mechanism essential for the initial round of Pol III transcription [45], is supported by *in vitro* data showing that TFIIIC is only required for assembling TFIIIB but is dispensable for Pol III transcription [46]. Other *in vitro* data suggest, however, that TFIIIC is not released from the DNA template once it is bound: pre-incubation of TFIIIC with one tRNA gene, followed by the addition of a second template as a competitor and then of all the other necessary components, led exclusively to transcription of the first gene [47].

Whether TFIIIC becomes displaced or disassembled during transcription initiation *in vivo* is currently unknown. Perhaps TFIIIC contacts the internal promoters even during Pol III elongation, as discussed below.

## 6. Recruitment of Pol III by TFIIIB and promoter melting

Among the three subunits of TFIIIB, only Bdp1 has no counterpart in the Pol II or Pol I transcription systems. Brf1 is a functional and structural analogue of TFIIB, and interacts with TBP and Bdp1 [48,49]. Despite the conserved TFIIB-like architecture, Brf1 harbours an additional functionality in its C-terminal extension. By C-terminal domain (CTD) Brf1 interacts with C34 subunit of Pol III and recruits Pol III to the transcription start site [50].

C34, C31 and C82 are the Pol III-specific subunits, which form a heterotrimer involved in Pol III initiation; the heterotrimer carries sequence motifs homologous to TFIIE, a general transcription factor of Pol II machinery. Additionally, Bdp1 has been reported to interact with C37, which together with C53 subunit forms a TFIIF-like subcomplex within Pol III [51]. In contrast with Pol II, where TFIIE and TFIIF participate in preinitiation complex formation by binding only transiently, the initiation of Pol III transcription is facilitated by its permanently bound TFIIF-like and TFIIE-like subcomplexes.

A major advance in understanding the unique features and peculiarities of the transcription initiation by *S. cerevisiae* Pol III has come from cryo-electron microscopy studies. The obtained structures showed two different conformations of the Pol III enzyme, allowing reconstruction of the two stages of the initiation process corresponding to the closed and open complexes. In the open conformation, the distance between the stalk and the C82–C34–C31 heterotrimer is smaller and a cleft is more open; therefore, the polymerase can better associate with the target DNA, whereas the closed conformation is similar to the structure of the elongating Pol III complex with a narrow cleft. Notably, even in the open conformation the cleft is narrower in Pol III than in other Pols [4].

The Pol III-specific subunit C34 contains winged helix (WH) domains by which it interacts with DNA and participates in DNA opening [48,52–55]. The promoter melting also involves the activity of C82, another Pol III-specific subunit which positions four WH domains on the clamp domain of the largest Pol III subunit C160 [4]. A rearrangement of two WH domains of C82 towards downstream DNA changes the orientation of the C82–C34–C31 subcomplex and remodels the active centre to produce the elongation complex [4].

## 7. Pol III elongation: uneven distribution of polymerase on transcription units

**Figure 3.**
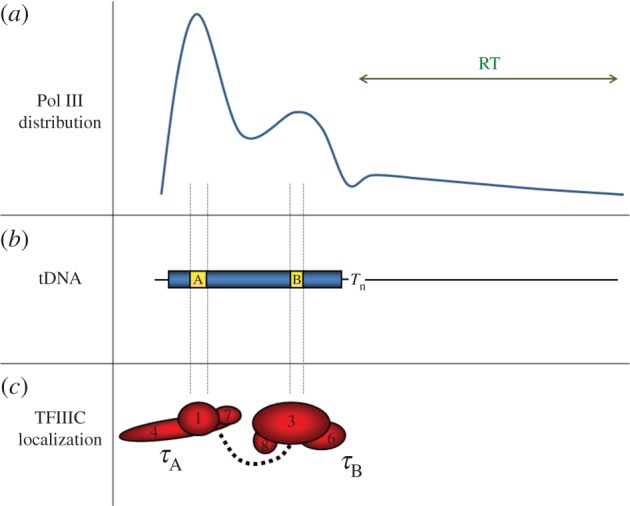
Uneven distribution of Pol III on transcription units. (*a*) Pol III distribution pattern, identified by CRAC method, across most genes, with a high peak of nascent transcript density over the 5′-end of the transcription unit and a weaker peak before the 3′-end of mature tRNA (intron-less tRNA gene is shown). Read-through (RT) of termination signal is observed on many tRNA genes, typically extending 50–200 nt beyond the expected canonical termination site. (*b*) Localization of A- and B-boxes of the bipartite internal promoter, and termination site (T*_n_*) in a tRNA-encoding gene (tDNA). (*c*) The τA and τB modules of TFIIIC factor binding the A- and B-boxes. Regions of postulated transient pausing of Pol III correspond to the TFIIIC binding sites.

Although Pol III genes are short, a recent genome-wide analysis of nascent transcripts attached to Pol III revealed a strikingly uneven polymerase distribution along the transcription units [36]. Inspection of individual tRNA genes showed a predominant pattern with high density of nascent transcripts over the 5′-end and a weaker peak before the 3′-end of the gene (figure 3). A minority of genes showed similar 5′ and 3′ peaks. Such uneven distribution of Pol III along the transcription unit suggests regional slow-down of elongation or transient pausing of the polymerase. Because on highly transcribed genes the 5′ peak is predominant, the initiation site clearance seems to be rate limiting during Pol III transcription. Interestingly, the 5′ and 3′ peaks of transcribing Pol III coincide, respectively, with the beginning of the A-box and of the B-box of the internal promoter (figure 3). The same was true for intron-containing genes, in which the distance between A-box and B-box is variable as they are separated by the intronic sequence. This suggests that TFIIIC bound to the A- and B-boxes could slow down the Pol III elongation rate leading to transient pausing.

While *in vitro* studies indicate that the TFIIIC–DNA interactions must be disrupted during Pol III elongation [56], a ChIP study [57] has revealed low but consistent TFIIIC occupancy at all transcriptionally active genes. The complex of TFIIIB and TFIIIC occupies a DNA length similar to that in nucleosomes, which are absent from tRNA genes [35,58]. Notably, the abundance of both τA and τB modules of TFIIIC on Pol III genes increases greatly during acute repression [59,60], indicating that transcription by Pol III partially displaces TFIIIC from its binding sites located in the transcribed region. This need to remove TFIIIC could explain the observed crowding of elongating Pol III exactly at the 5′ borders of the A- and B-boxes bound by this transcription factor.

## 8. Transcription termination and read-through of termination signal

Several excellent reviews about Pol III termination have been recently published [61–64], so here this topic will be described only briefly.

The signal for Pol III termination is an oligo(T) track in the non-transcribed DNA strand [65–67]. In humans, four thymidines are sufficient for Pol III termination, in *Schizosaccharomyces pombe* five and in *S. cerevisiae* six [66,67]. Furthermore, both *in vitro* and *in vivo* data show a correlation of termination efficiency by yeast Pol III with the length of the oligo(T) tract [36,68].

Three subunits of Pol III are important for termination: C53, C37 and C11. C53 and C37 form a heterodimer and are engaged in the recognition of the termination signal [69], while C11 is required for RNA cleavage [70–72]. Pol IIIΔ (lacking C11, C37 and C53) terminates on oligo(T) less efficiently than the wild-type enzyme because of an increased elongation rate; addition of the C53-C37 subcomplex reduces the global elongating rate and corrects the terminator recognition defect of Pol IIIΔ [69,72–74].

The C53–C37 subcomplex dissociates easily from Pol III and has been detected in the free form [12]. In the Pol III structure, it sits across the cleft, near the presumed location of downstream DNA [75], and the residues involved in transcription termination position close to the non-template DNA strand [4]. The C37 subunit extends towards the DNA-binding cleft where its flexible loop contacts the C34 subunit involved in transcription initiation and the TFIIIB subunit Bdp1 [4]. The C-terminal part of C37 was localized within the Pol III active centre [4,51,76] and its deletion leads to a loss of subunits C11 and C53 upon purification [69].

The C11 subunit is composed of two domains, both crucial for Pol III functioning; the N-terminal domain is homologous to Rpb9, a subunit of Pol II, and the C-terminal one shows homology to the TFIIS factor of Pol II [70]. The N-terminal domain is required for terminator recognition and pausing [77] as well as transcription reinitiation [69]. Structure analysis has shown that it is mobile, located next to the C53–C37 subcomplex and only temporarily recruited to the catalytic centre [4,75]. The C-terminal TFIIS-homologous domain in C11 is responsible for 3′ RNA cleavage that occurs during terminator pausing [69,77]. The function of the C-terminal domain of C11 in Pol III termination was supported by experiments *in vivo* exploiting C11 point mutants [74].

In *in vitro* experiments wild-type Pol III from *S. cerevisiae* terminated efficiently on 7T and 9T terminators, while Pol IIIΔ recognized only the 9T terminator [72]. This prompted the authors to propose two mechanisms of Pol III termination [72]. The core mechanism would be C57–C37- and C11-independent and would require at least eight thymidines for destabilization of oligo(rU·dA) heteroduplex and efficient termination, while a holoenzyme mechanism operating in the presence of the C53–C37 heterodimer and C11 subunit would also recognize short oligo(T) tracks. The core mechanism autonomously destabilizes the complete Pol III elongating complex [72–74], leaving Pol IIIΔ terminator-arrested [72]. The authors suspected that the terminator arrest involves backtracking. A role of Pol III backtracking in termination has also been suggested by an independent study [78]. Supplementation by cleavage activity of the C11 subunit rescues the backtracked Pol IIIΔ; however, to prevent terminator arrest, the C11 subunit cooperates with the N-terminal domain of C53 [72]. The holoenzyme termination mechanism is based on slowing down elongation on the oligo(A) track in the template strand and preventing terminator arrest [72]. Further analysis has revealed that formation of a metastable pre-termination complex (PTC) is required for transcript release by Pol III [73]. To convert the elongation complex to the PTC, Pol III subunits C53, C37 and C11 act together with the third and fourth T residues of the non-template strand. Then the C-terminal region of C37 and T5 of the non-template strand contribute to transcript release [73]. Cryo-EM structural data have confirmed these results—five amino acid residues from the flexible loop in the C37 subunit interact with the first four thymidines of the non-template DNA strand to effect a switch towards PTC, while the fifth (thymidine) brings about transcription termination [4]. All these data show that Pol III termination, which looks quite simple at first, is more complicated when studied in detail.

Another initially unanticipated aspect of Pol III termination is read-through (RT) of the termination signal. RT of terminator signal is quite common in human cells [79]. Several reports have described terminator RT *in vivo* in Pol III termination mutants in the C11 and C37 subunits in *S. pombe* [74,77,80]; however no RT of 8T terminator has been observed in a wild-type strain [74]. Other *in vitro* experiments showed that the strength of 5T terminator in *S. cerevisiae* depends on the sequence downstream of the terminator; a CT sequence acts as a weakening element, while an A or G following the terminator increases its efficiency [67]. Notably, studies in human cells showed that Pol III occupies the region downstream from the 3′-end of many tRNA genes [37,79]. Additionally, downstream nucleosome mobility towards tRNA gene may inhibit transcription by restricting the access of Pol III to the gene terminator. Under a repressed state, a downstream nucleosome shows mobility towards tRNA gene [58]. This is consistent with our recent data indicating reduced transcription for nearly all tRNAs under stress conditions [36]. A recent genome-wide analysis of active Pol III in *S. cerevisiae* confirmed effective termination on 7T and 8T tracts [36]. Importantly, this analysis showed substantial RT of termination signal on many tRNA genes, typically extending 50–200 nt beyond the expected terminator (figure 3). The presence of 3′-extended Pol III transcripts was confirmed by Northern blotting, but these extended transcripts were rapidly processed or degraded. An average RT level for all tRNA genes was about 10%, but reached over 40% for some tRNA genes [36]. Termination generally occurs at the canonical terminator, but its efficiency is highly variable and RT levels were negatively associated with the oligo(T) length: for genes with more than 25% RT, more than 60% have 6T tracts as the longest termination signal, whereas for genes with less than 5% RT, 60% have 8T tracts. RT levels *in vivo* show additional correlation with uracil abundance in the 3′-extended tRNA transcripts but not with the sequence directly downstream of the terminator [36]. Independent studies identified long RT of termination signal in tRNA genes of *S. pombe* mutant lacking a mediator complex subunit, Med20 [81]. These extended transcripts were polyadenylated and targeted for degradation by the exosome. Is seems therefore that the RT of the termination signal is a feature of Pol III in many organisms.

Recent studies have revealed that proteins involved in mRNA biogenesis are important in regulation of Pol III transcription. Nab2 protein, known as nuclear polyadenylated RNA-binding protein, required for maturation and export of mRNA, interacts with Pol III, TFIIIB and Pol III transcripts. During transcription elongation, Nab2 remains associated with Pol III and/or the nascent transcript, and may also participate in surveillance of 3′ extended pre-tRNAs [36,82]. Moreover, experiments in fission yeast have shown that Swd2.2 and Sen1 act directly at Pol III-transcribed genes to limit the association of condensin. It was shown that at least active Pol III transcription is not an obstacle for the binding of condensin [83]. However, in *S. cerevisiae*, Sen1 was not identified as a Pol III-interacting protein [84].

## 9. Regulation of Pol III by Maf1

Pol III is specifically regulated by a global negative effector Maf1, originally identified in *Saccharomyces cerevisiae* by a classical genetic approach [85]. One of the yeast mutants selected in a screen for tRNA-mediated suppressors accumulated high tRNA levels and additionally had a growth defect. That allowed cloning of the gene for yeast Maf1, which turned out to be the founding member of a new class of Pol III negative effectors [86]. Several research groups showed that, apart from yeast, Maf1 orthologues function as Pol III repressors in mammals, flies, worms, plants and parasites [87–91]. Maf1 proteins from diverse organisms share N- and C-terminal regions of homology.

Maf1 is targeted by several signalling pathways modulating its phosphorylation status and thereby mediates various stress signals to Pol III [92]. Under favourable growth conditions, Maf1 is phosphorylated and in this form is localized to the cytoplasm. Upon a shift to repressive conditions, Maf1 is dephosphorylated and imported to the nucleus, where it binds directly to the Pol III complex, preventing Pol III-directed transcription [60,93].

Analysis of the Pol III structure in complex with Maf1 [55] showed that Maf1 binds to the Pol III clamp at the rim of the cleft and re-arranges the structure of the C82–C34–C31 trimer over the active centre. By relocating a specific WH domain of the C34 subunit, Maf1 weakens the interaction of C34 with the Brf1 subunit of the TFIIIB initiation factor and thereby impairs Pol III recruitment to promoters [55,94].

Exactly how Maf1 is recruited to Pol III during ongoing transcription is unknown. Maf1 does not bind to a preassembled Pol III–Brf1–TBP–DNA initiation complex and the interactions of Pol III with Maf1 and Brf1-TBT-DNA are mutually exclusive [55,95]. Significantly, Maf1 does not impair Pol III elongation to the end of the template [55]. No effect of Maf1 on the Pol III distribution along the transcription units has been detected either [36].

It is noteworthy that Maf1 alone is not sufficient to repress Pol III which is also directly regulated by posttranslational modifications of its specific subunits: Pol III is repressed by phosphorylation of C53 whereas sumoylation of C82 leads to Pol III activation [22,96]. Moreover, Pol III is also regulated by differential phosphorylation of Bdp1 subunit of TFIIIB transcription factor [97].

The rate of Pol III transcription increases at least fivefold through a process known as facilitated recycling, which couples the termination of transcription with reinitiation [98]. An accepted model assumes Maf1 binding to the Pol III elongation complex at each transcription cycle and its dissociation prior to the initiation of the next cycle [99]. CK2 kinase, which is associated with the Pol III-containing chromatin, ensures a high rate of transcription through phosphorylation of Maf1, TFIIIB and potentially also other Pol III components [100,101]. Conversely, when cells encounter unfavourable growth conditions, the CK2 catalytic subunit dissociates from the Pol III complex and is no longer able to stimulate transcription. Moreover, dephosphorylated Maf1 is imported from the cytoplasm increasing its concentration in the nucleus. This is the time when Maf1 takes over control and inhibits transcription. This mechanism ensures constant monitoring of the environment and a transcription shut-down immediately after the conditions become adverse.

**Figure 4.**
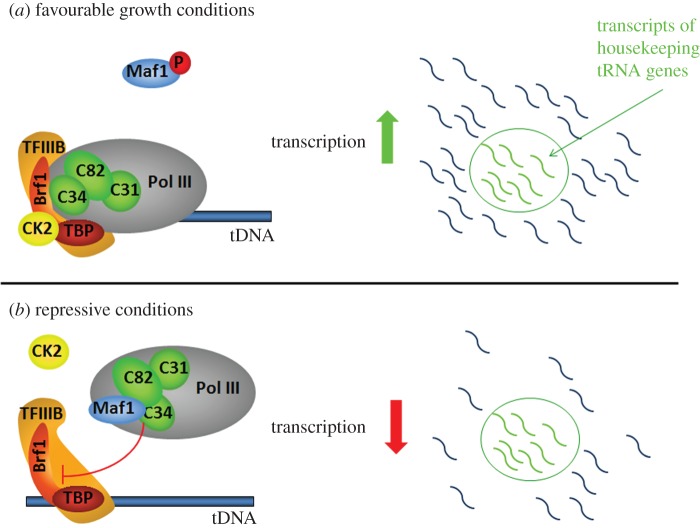
Regulation of Pol III transcription by Maf1. (*a*) Under favourable growth conditions, Maf1 is inactivated by phosphorylation. CK2 kinase phosphorylates Maf1 and also TFIIIB initiation factor associated in the promotor region stimulating Pol III transcription. (*b*) Upon shift to repressive conditions, CK2 dissociates from the Pol III complex. Dephosphorylated Maf1 binds directly to Pol III complex and weakens interaction of C34 with the Brf1 subunit of the TFIIIB initiation factor, and thereby impairs Pol III recruitment to promoters reducing transcription for nearly all tRNA genes. However, a subset of housekeeping tRNA genes marked in green exhibits low responsiveness to Maf1.

Interestingly, Maf1 regulates the levels of different tRNAs to various extents [102,103]. Recently, relative transcription intensity by Pol III was compared over all nuclear tRNA genes under near optimal growth conditions and following transfer to stress conditions known to repress tRNA expression. Although under stress conditions reduced transcription was observed for nearly all tRNAs, the degree of the repression was highly variable among the tRNA genes, a subset of tRNA genes being markedly less repressed [36] (figure 4). This conclusion is broadly consistent with a previous microarray analysis which revealed that the levels of mature tRNAs were reduced to variable extents by stress conditions [102]. Similarly, Pol III shows different enrichment on isogenes and indicates different transcriptional activity on gene copies within family. Additionally, in wild-type strain tRNA levels are different across the families, and show different response to starvation [58]. The heterogeneity in the tRNA repression seen in the wild-type is substantially reduced in a mutant lacking Maf1. This provides genome-wide evidence that Maf1 does not simply down-regulate all tRNAs, but affords an additional layer of gene-specific Pol III regulation. A subset of tRNA genes shows low responsiveness to both environmental and cellular signals. Notably, this group contains at least one tRNA for each amino acid. Together these findings suggest the existence of a basal subset of housekeeping tRNA genes [36]. This concept is consistent with the mode of Maf1-mediated repression of actively transcribed tRNA genes in human cells subjected to serum starvation [104].

## 10. Perspectives

The past decade has seen substantial progress in delineating the mechanisms by which Pol III-mediated tRNA gene transcription is controlled. The unique features of Pol III that distinguish it from other polymerases and novel insights on its functional characteristics have been incorporated in and also draw from atomic models of Pol III in different conformations. However, the mode of Pol III interaction with general negative regulator Maf1 is known in outline only and the mechanism of the repression deserves future studies. The differential specificity of Maf1 towards various genes probably relies on additional factors interacting with Pol III chromatin, which need to be elucidated.

Findings regarding the Pol II system have revealed that much transcription regulation occurs after recruitment of the polymerase to promoter through controlling pausing and elongation. Recently, pausing of Pol III has been documented by mapping of the transcriptionally active enzyme at nucleotide resolution [36]. The intriguing hypothesis that the pausing and elongation of Pol III is controlled by association of TFIIIC factor with internal promoter sequences should be validated experimentally. Pol II uniquely employs the so-called mediator complex and carries an extra CTD on its largest subunit, Rpb1. The CTD undergoes dynamic phosphorylation during the progression from initiation through elongation to termination and transcription arrest triggers Rpb1 ubiquitination [105,106]. Although the largest Pol III subunit has no CTD, several components of Pol III apparatus undergo phosphorylation or sumoylation, and ubiquitination is also considered [22,96,97,107,108]. Nothing is known, however, about a possible involvement of phosphorylation, or any other as yet unknown type of modification in the progression of different stages of Pol III transcription. Possibly unknown modifications of Pol III component exist that mark the stage of transcription cycle.
